# Psoriasis Is Associated With an Increased Risk of Hospitalization for Systemic Lupus Erythematosus: Analysis of the National Inpatient Sample Database

**DOI:** 10.7759/cureus.11771

**Published:** 2020-11-29

**Authors:** Pius E Ojemolon, Chinedu E Unadike, Fidelis Uwumiro

**Affiliations:** 1 Anatomical Sciences, St. George's University, St. George's, GRD; 2 General Surgery, Queen Elizabeth Hospital NHS Trust, King's Lynn, GBR; 3 Internal Medicine, Our Lady of Apostles Hospital, Akwanga, NGA

**Keywords:** psoriasis, lupus, cutaneous manifestations of systemic disease, sle, hospitalization, national inpatient sample, large-database, dermatology, rheumatology

## Abstract

Background: There is a scarcity of literature on co-existing psoriasis (Ps) and systemic lupus erythematosus (SLE). We used a large national population database to determine if there is any association between Ps and SLE. The primary objective was to compare the odds of being admitted for SLE in patients with Ps compared to those without Ps. The secondary objective was to compare hospital outcomes of patients admitted for SLE with co-existing Ps to those without Ps.

Methods: Data were abstracted from the National Inpatient Sample (NIS) 2016 and 2017 Databases. We search for hospitalizations using ICD-10 codes. Multivariate logistic and linear regression analysis was used accordingly to adjust for confounders.

Results: There were over 71 million discharges included in the database. A total of 20,630 hospitalizations had SLE as the principal diagnosis. One hundred fifty (0.7%) of these SLE hospitalizations have co-existing Ps. Hospitalizations for SLE with co-existing Ps had similar length of stay (LOS), total hospital charges, need for blood transfusion, odds of having a secondary discharge diagnosis of venous thrombosis or embolism/pulmonary embolus, and acute kidney injury compared to those without Ps. Hospitalizations with a secondary diagnosis of Ps have an adjusted odds ratio (AOR)=2.73 (95% CI 1.86-4.02, P<0.0001) of SLE being the principal reason for hospitalization compared to hospitalizations without Ps.

Conclusion: In our study, patients with Ps had almost three times the odds of being admitted for SLE compared to non-Ps patients. However, Ps patients admitted for SLE had similar hospital outcomes compared to non-Ps patients admitted for SLE.

## Introduction

Psoriasis (Ps) and systemic lupus erythematosus (SLE) are two rheumatologic conditions that typically present with cutaneous manifestations but are both characterized by systemic inflammation and a pronounced autoimmune background [1,2]. Both diseases are driven by immune dysfunction and are associated with extra-cutaneous manifestations, with arthritis being common to both. Similar to SLE, psoriasis is an autoimmune disease with associated medical co-morbidities that increase the risk of hospitalization [3-6].

In most developed countries, Ps affects between 1.5 and 5% of the populace, while the reported prevalence of SLE in the United States (US) ranges from 40 to 271 cases per 100,000 persons [7]. The health care burden of these conditions goes beyond the physical dimensions of disease. Ps and SLE have extensive physical, psychosocial, and emotional effects on patients, and can result in stigmatization and poor self-esteem which affects social functioning and interpersonal relationships, reducing the quality of life. Arthritis, often a feature of both diseases, is one of the largest global contributors to patient disability. Both diseases additionally impart an overall increased risk of mortality compared to the general population [8,9].

There is a scarcity of literature on co-existing Ps and SLE. Studies are limited to case series and small, single-center retrospective studies. These studies show similarities in the pathogenesis of both diseases, with the prevalence of Ps in SLE patients reportedly being between 0.5 and 2.5%, depending on the patient population [10-13]. To bridge this knowledge gap, we used a large national population database to determine if there any association exists between Ps and SLE, and secondarily compared outcomes between patients admitted for SLE with and without co-existing Ps.

## Materials and methods

Source of data

We carried out a retrospective study of hospitalizations in 2016 and 2017 with a principal diagnosis of SLE with and without a secondary diagnosis of Ps. Hospitalizations were obtained from the National Inpatient Sample (NIS) database. The NIS is under the jurisdiction of the Agency for Healthcare Research and Quality [14]. NIS is the largest inpatient national database in the US [15,16]. Discharges are weighted to maintain national representation [17]. A maximum of 30 and 40 discharge diagnoses per hospitalization can be recorded in 2016 and 2017 NIS, respectively [18]. Diagnoses are recorded using ICD-10 (International Classification of Disease) codes (see appendix). The main diagnosis responsible for admission is the primary diagnosis. All other diagnoses are secondary diagnoses [19]. NIS database does not contain identifying inpatient information. Hence, we waived institutional review board approval. This section is similar to prior NIS papers [14-19].

Exclusion criteria

Nonadult hospitalizations for patients less than 18 years were excluded. Baseline characteristics of SLE hospitalization with and without a secondary diagnosis of Ps were compared using chi-square test.

Outcomes

Primary Objective

Compare the odds of SLE being the principal diagnosis for hospitalizations with and without a secondary diagnosis of Ps.

Secondary Objective

Compare outcomes of hospitalizations for SLE with and without a secondary diagnosis of Ps. Outcomes of interest were inpatient mortality, similar length of stay (LOS), total hospital charges, need for blood transfusion, odds of having a secondary discharge diagnosis of venous thrombosis or embolism (VT)/pulmonary embolus (PE), and acute kidney injury (AKI).

Statistical Analysis

Analyses were performed using statistics and data (STATA, version 16, StataCorp., College Station, TX, USA).

Primary objective

Univariate logistic regression analysis using all variables and co-morbidities in Table 1 was used to calculate unadjusted odds ratios (ORs) for SLE being the principal diagnosis for hospitalizations with and without a secondary diagnosis of Ps. Co-morbidities were selected from a review of the literature. Charleston index was used to adjust for comorbidity burden. All variables with P-values <0.1 were included in a multivariate logistic regression model which was used to calculate the adjusted odds ratio of SLE being the principal diagnosis for hospitalizations with and without a secondary diagnosis of Ps. Values of p<0.05 were considered significant in the multivariate analysis.

**Table 1:** Baseline characteristics of SLE hospitalizations with and without psoriasis SLE: Systemic Lupus Erythematosus, Ps: Psoriasis, MI: Myocardial infarction, COPD: Chronic obstructive pulmonary disease, DM: Diabetes Mellitus, CHF: Chronic congestive heart failure, CKD: Chronic kidney disease, O2: oxygen, median household income: median household income for patient’s Zip code. *: Stata can not compute p-value because one of the subpopulations is zero. **:Secondary diagnoses

Baseline Characteristics	SLE without Ps (n=20,480)	SLE with Ps (n=150)	p-value
Mean age (years)	37.8	41.9	0.209
Female gender	86.60%	80.00%	0.2863
Race			0.0005
White	23.00%	53.30%	Reference
Black	46.60%	30.00%	0.002
Hispanic	20.30%	10.00%	0.014
Asians	4.90%	3.30%	0.233
Native Americans	0.50%	3.30%	0.252
Others	4.70%	0	*
Charleston comorbidity index			0.1991
1	44.00%	40.00%	
2	17.60%	30.00%	
≥3	38.30%	30.00%	
Hospital bed size			0.5569
Small	13.70%	20.00%	
Medium	24.80%	20.00%	
Large	61.60%	60.00%	
Hospital teaching status			0.3318
Nonteaching	19.60%	26.70%	
Teaching	80.40%	73.30%	
Hospital location			0.2996
Rural	3.30%	6.70%	
Urban	96.70%	93.30%	
Expected primary payer			0.163
Medicare	25.60%	37.90%	
Medicaid	35.40%	41.40%	
Private	32.50%	20.70%	
Self-pay	6.60%	0%	
Median household income(quartile)			0.0162
1^st ^(0-25^th^)	39.40%	41.40%	
2^nd ^(26th-50^th^)	23.80%	41.40%	
3^rd^ (51st-75^th^)	21.70%	0%	
4^th^ (76th-100^th^)	15.20%	17.20%	
Hospital region			0.5759
Northeast	20.60%	20.00%	
Midwest	17.20%	26.70%	
South	43.20%	36.70%	
West	19.10%	16.70%	
Comorbidities**			
Dyslipidemia	12.60%	6.70%	0.3318
Old MI	2.30%	3.30%	0.6982
Atrial fibrillation	2.70%	3.30%	0.8197
COPD	4.50%	6.70%	0.5674
Hypertension	29.50%	20.00%	0.2516
Hypothyroidism	10.30%	16.70%	0.2557
DM type	10.00%	10.00%	0.995
CHF	11.70%	10.00%	0.776
CKD	28.80%	23.30%	0.5071
Liver disease	5.00%	10.00%	0.2138
Electrolyte derangement	34.20%	26.70%	0.3856
Maintenance hemodialysis	7.80%	6.70%	0.8146
O2 dependence	1.40%	3.30%	0.3578
Smoking	11.70%	16.70%	0.394
Anemia	59.60%	40.00%	0.0298

Secondary objective

Multivariate logistic regression for categorical outcomes and linear regression analysis for continuous outcomes, using all variables and co-morbidities in Table 1 were used to adjust for confounders for outcomes of hospitalizations for SLE with and without a secondary diagnosis of Ps. All p-values were 2-sided, with 0.05 as the threshold for statistical significance.

## Results

There were over 71 million discharges included in the database. Hospitalizations for adult patients age ≥18 years were included. 320,610 hospitalizations had Ps as a secondary diagnosis. 20,630 hospitalizations had SLE as principal diagnosis. One hundred fifty (0.7%) of these SLE hospitalizations have Ps as a secondary diagnosis, while 20,480 (99.3%) did not have Ps as a secondary diagnosis. Characteristics of hospitalizations with SLE as principal diagnosis with and without a secondary diagnosis of Ps are displayed in Table 1.

Ps group had more Caucasians, and less anemia compared to the non-Ps group. From all hospitalizations with SLE as principal diagnosis, 255 (1.2%) resulted in inpatient mortality. Two hundred fifty-five of these deaths occurred in hospitalizations without Ps as a secondary diagnosis. Hospitalizations for SLE with co-existing Ps had similar LOS, total hospital charges, need for blood transfusion, odds of having a secondary diagnosis of VT/PE and AKI compared to those without Ps (Table 2). 

**Table 2:** Univariate association of baseline variables with the primary objective SLE: Systemic Lupus Erythematosus, MI: Myocardial infarction, PCI: percutaneous coronary intervention, COPD: Chronic obstructive pulmonary disease, DM: Diabetes Mellitus, CHF: Chronic congestive heart failure, CKD: Chronic kidney disease, O2: oxygen, median household income: median household income for patient’s Zip code, * included in multivariate logistic regression analysis

Baseline variables	Odds ratio	p-value
Age*	0.99	<0.0001
Female gender*	4.96	<0.0001
Race		
White*	Reference	Reference
Black	8.52	<0.0001
Hispanic	4.58	<0.0001
Asians	4.45	<0.0001
Native Americans	2.18	0.002
Others	3.74	<0.0001
Charleston comorbidity index*	1.13	<0.0001
Hospital bed size*		
Small	Reference	Reference
Medium	1.19	0.004
Large	1.68	<0.0001
Hospital teaching status*		
Non-teaching	Reference	Reference
Teaching	2.00	<0.0001
Hospital location*		
Rural	Reference	Reference
Urban	2.89	<0.0001
Expected primary payer*		
Medicare	Reference	Reference
Medicaid	2.39	<0.0001
Private	1.71	<0.0001
Self-pay	2.63	<0.0001
Median household income(quartile)*		
1^st^ (0-25^th^)	Reference	Reference
2^nd^ (26^th^-50^th^)	0.71	<0.0001
3^rd^ (51^st^-75^th^)	0.70	<0.0001
4^th^ (76^th^-100^th^)	0.59	<0.0001
Hospital region*		
Northeast	Reference	Reference
Midwest	0.69	<0.0001
South	0.98	0.785
West	0.85	0.018
Dyslipidemia*	0.39	<0.0001
Old MI*	0.48	<0.0001
Old PCI*	0.25	0.002
Old pacemaker*	0.21	<0.0001
Atrial fibrillation*	0.29	<0.0001
COPD*	0.34	<0.0001
Old stroke	1.04	0.546
Hypertension	0.96	0.356
Peripheral vessel disease*	0.29	<0.0001
Hypothyroidism	1.02	0.681
DM*	0.40	<0.0001
Obesity*	1.10	0.038
CHF	0.94	0.186
CKD*	2.56	<0.0001
Liver disease*	1.26	0.002
Electrolyte derangement*	2.25	<0.0001
Maintenance hemodialysis*	3.79	<0.0001
O2 dependence*	0.48	<0.0001
Smoking*	0.62	<0.0001
Anemia*	4.33	<0.0001

Univariate association of variables and co-morbidities with the odds of SLE being the principal reason for hospitalization, highlighting the variables included in the multivariable logistic regression model are displayed in Table 3. Hospitalizations with a secondary diagnosis of Ps have an adjusted odds ratio (AOR)=2.73 (95% CI 1.86-4.02, P<0.0001) of SLE being the principal reason for hospitalization compared to hospitalizations without Ps.

**Table 3:** Hospital outcomes of SLE hospitalizations with and without Psoriasis SLE: Systemic Lupus Erythematosus, Ps: Psoriasis, LOS: Hospital length of stay, C.I: Confidence Interval, OR: Odds Ratio, USD: United states dollars. * Stata cannot compute statistics if one of the subpopulations is less than 10, VT: Venous thrombosis or embolism, PE: Pulmonary embolus, AKI: Acute kidney injury

Hospital outcomes	SLE with Ps (n=150)	SLE without Ps (n=20,480)	Adjusted OR (95% CI)	p-value
No. of in hospital deaths	0	255	*	*
VT/PE	3.3	4.4	0.78 (0.09-6.64)	0.820
Transfusion	10.0	9.8	1.70 (0.49-5.81)	0.403
AKI	23.3	26.6	1.13 (0.35-3.62)	0.841
			Adjusted mean difference	
LOS, mean, days	5.6	6.6	-0.15 ({-1.36}-1.07)	0.815
Total charges, mean USD	50,636	67,248	-2036 ({-18,126}-14,054)	0.804

## Discussion

The prevalence of Ps in the general US population has been estimated to be about 1-3% [20]. Although only 0.7% of hospitalizations for SLE had co-existing Ps in our study, patients with Ps had almost three times the odds of being admitted for SLE compared to non-Ps patients. A retrospective study of 445 SLE patients attending an academic rheumatology clinic conducted by Bonilla et al. found a 5.1% prevalence of Ps, which was significantly higher than that observed in the general population [13].

The factors involved in the pathogenesis of concomitant Ps and SLE are not clearly understood but are thought to revolve around the balance of the major T helper lymphocyte subset populations (Th1, Th2, and Th17). SLE is largely thought to involve an apparent shift of this balance towards Th2 immune responses leading to B cell hyperactivity and production of autoantibodies. Ps, on the other hand, is powered by a predominantly Th1 immune response with elevated amounts of Th1 pathway cytokines (IFN-γ and IL-2) demonstrated in several Ps patients. However, the upregulation of the Th17 immune pathway is a mechanism shared by both diseases. IL-17, IL-22, and IL-23, produced by Th17 cells, have been shown to play a major role in maintaining chronic inflammation in both psoriasis and SLE, and an upregulated Th17 immunologic response may explain the association between both conditions, as well as provide a therapeutic target for management of concomitant Ps and SLE [8,12,20-22].

The mean ages of patients in our study were 42 and 38 years for the SLE with and without Ps cohorts respectively, and the majority of patients in both cohorts were female. Regarding the racial distribution, more than half of the patients with SLE and Ps were White, while most of the patients in the SLE without Ps cohort were Black. These are in keeping with the findings of studies that suggest that Ps and SLE are most prevalent amongst middle-aged patients, with SLE particularly more common in African-American females while Ps typically affects White patients [7-8,22-24].

A multiplicity of studies has demonstrated that both Ps and SLE patients have increased traditional cardiovascular risk factors compared to the general population, with systemic inflammation and inflammatory pathways earmarked as a major contributor [25]. A large prospective cohort study carried out by Khalid et al. demonstrated a significant dose-dependent relationship between psoriasis skin disease severity and new-onset clinical heart failure (HF), with hazard ratios (HR) of 1.22 (95% CI = 1.16-1.29) for mild disease and 1.53 (95% CI = 1.34-1.74) for the severe disease [26], while a similar study conducted by Chen et al. among Medicaid patients using data from 2007 to 2010 showed that multivariable-adjusted HRs for HF was 2.7 (95% CI = 2.3-3.1) for SLE patients compared to the general Medicaid population [27]. Our study did not demonstrate an increase in cardiovascular risk factors such as hypertension, dyslipidemia, old myocardial infarction, atrial fibrillation, smoking history, and congestive heart failure in the SLE-Ps cohort compared to the SLE-no Ps cohort. There also was no significant difference in the odds of autoimmune-related disorders such as hypothyroidism between both groups. Our study additionally showed that both cohorts exhibited no significant difference in the distribution of CKD, liver disease, electrolyte derangement, or being on maintenance dialysis.

Besides reaching an accurate diagnosis, a major difficulty in patients with co-existing Ps and SLE is to provide proper treatment. Therapies that may be useful in one condition may be contraindicated in the other. For example, the use of systemic corticosteroids, which is commonplace for management of SLE, may have a negative effect on the course of severe Ps as it increases the risk of conversion to pustular Ps, whereas phototherapy, which is widely utilized in Ps, may cause exacerbation of the cutaneous manifestations of SLE [28]. Immune modulators and biologic therapies have become increasingly useful in cases of co-existing Ps and SLE. Medications targeting immunologic pathways common to both diseases such as methotrexate, tumor necrosis factor-alpha (TNF-α) inhibitors, ustekinumab (which specifically targets the Th17 signaling pathway), and abatacept have been shown to have promising effects in patients with both diseases [12,28].

Our study highlighted that Ps patients admitted for SLE had similar LOS and total hospital charges compared to non-Ps patients admitted for SLE. Both patient groups also had similar odds of in-hospital VT/PE, AKI, and need for blood transfusion. These findings show that co-existing Ps do not worsen SLE hospitalization outcomes. This may be reflective of an improved understanding of the shared immunologic pathways and expanded treatment options available to combat concomitant SLE and Ps.

Our study has several strengths. First, we utilized data from the NIS, a large nationwide database to provide a large sample size. This large sample size significantly increases the power of our study. Second, the NIS database allows us to compare baseline demographic characteristics and hospital events/outcomes between SLE hospitalizations with and without concomitant Ps. Our study has some limitations. First, we cannot determine if Ps preceded or followed the diagnosis of SLE. Hence causation can not be determined from this study, but rather, a potential association between Ps and SLE. Second, the NIS database which is based on ICD-10 codes uses “claims data” for billing purposes rather than “clinical data”, hence errors may occur using ICD-10 codes [29]. Third, this report reflects data on hospitalizations rather than on individual patients [30]. Therefore, individuals with multiple hospitalizations will be counted multiple times. Fourth, we were unable to sub-classify Ps by severity and duration. Lastly, we are unable to determine the effect of treatment and medication adherence on outcomes. Further studies on the mechanism by which Ps increases the odds of SLE hospitalizations are needed.

## Conclusions

Patients with Ps had almost three times the odds of being admitted for SLE compared to non-Ps patients in our study. However, Ps patients hospitalized for SLE had similar in-hospital outcomes compared to non-Ps patients hospitalized for SLE. Further studies are needed to study this potential association.

## Appendices

Used ICD-10 codes

Psoriasis

L40

SLE

M32

Dyslipidemia

E78

Old MI

I252

Atrial fibrillation

I480, I481, I482, I4891

Chronic obstructive pulmonary disease

J41, J42, J43, J44

Hypertension

I10

Hypothyroidism

E03

Diabetes Mellitus Type 1&2

E10, E11

Congestive heart Failure

I50

Chronic Kidney Disease

N18

Liver disease

K70, K71, K72, K73, K74, K75, K76, K77

Electrolyte derangement

E870, E871, E872, E873, E874, E875, E876

Maintenance dialysis

Z992

Oxygen dependence

Z9981

Smoking

Z87891, F17200

Anemia

D50, D51, D52, D53, D55, D56, D57, D58, D59, D60, D61, D62, D63, D64

Hospital outcomes

Blood transfusion

302

VT

I82

PE

I26

AKI

N17

Abbreviations:

SLE: Systemic Lupus Erythematosus, MI: Myocardial infarction, VT: Venous thrombosis/embolism, PE: Pulmonary Embolus, AKI: Acute Kidney Injury

## References

[1] Hawkes JE, Chan TC, Krueger JG (2017). Psoriasis pathogenesis and the development of novel targeted immune therapies. J Allergy Clin Immunol.

[2] Kaul A, Gordon C, Crow MK (2016). Systemic lupus erythematosus. Nat Rev Dis Primers.

[3] Edigin E, Eseaton PO (2020). LB932 Reasons for psoriasis hospitalizations with inpatient mortality. J Investig Dermatol.

[4] Edigin E, Rivera Palon MM (2020). LB934 Outcomes of psoriasis with and without joint involvement. J Investig Dermatol.

[5] Korman NJ (2020). Management of psoriasis as a systemic disease: what is the evidence?. Br J Dermatol.

[6] González LA, Alarcón GS (2017). The evolving concept of SLE comorbidities. Expert Rev Clin Immunol.

[7] (2018). Centers for Disease Control and Prevention. Systemic lupus erythematosus. https://www.cdc.gov/lupus/facts/detailed.html.

[8] Kim WB, Jerome D, Yeung J (2017). Diagnosis and management of psoriasis. Can Fam Physician.

[9] Yeo S, Dias S, Isenberg D (2018). Advances in systemic lupus erythematosus. Medicine.

[10] Millns JL, Muller SA (1980). The coexistence of psoriasis and lupus erythematosus: an analysis of 27 cases. Arch Dermatol.

[11] Tselios K, Yap KS, Pakchotanon R, Polachek A, Su J, Urowitz MB, Gladman DD (2017). Psoriasis in systemic lupus erythematosus: a single-center experience. Clin Rheumatol.

[12] Tan JS, Tababa EL, Dimacali CD, Yap-Silva C (2017). Systemic lupus erythematosus with coexistent psoriasis vulgaris: a case report. Acta Medica Philippina.

[13] Bonilla E, Shadakshari A, Perl A (2016). Association of psoriasis and psoriatic arthritis with systemic lupus erythematosus. Rheumatol Orthop Med.

[14] Edigin E, Eseaton P, Kaul S (2020). Systemic sclerosis is not associated with worse outcomes of patients admitted for ischemic stroke: analysis of the national inpatient sample. Cureus.

[15] Edigin E, Ojemolon PE, Eseaton PO, Shaka H, Akuna E, Asemota IR, Manadan A (2020). Rheumatoid arthritis patients have better outcomes when hospitalized for ischemic stroke: analysis of the National Inpatient Sample [published online ahead of print, 2020 Sep 10]. J Clin Rheumatol.

[16] Edigin E, Akuna E, Asemota I, Eseaton P, Ojemolon PE, Shaka H, Manadan A (2020). Rheumatoid arthritis does not negatively impact outcomes of patients admitted for atrial fibrillation. Cureus.

[17] Edigin E, Shaka H, Eseaton P (2020). Rheumatoid arthritis is not associated with increased inpatient mortality in patients admitted for acute coronary syndrome. Cureus.

[18] Edigin E, Ojemolon PE, Eseaton PO, Shaka H, Akuna E, Asemota IR, Augustine M (2020). Systemic sclerosis is associated with increased inpatient mortality in patients admitted for atrial fibrillation: analysis of the National Inpatient Sample [published online ahead of print, 2020 Sep 16]. J Clin Rheumatol.

[19] Ojemolon PE, Shaka H, Edigin E (2020). Impact of diabetes mellitus on outcomes of patients with knee osteoarthritis who underwent knee arthroplasty: an analysis of the Nationwide Inpatient Sample. Cureus.

[20] Huerta C, Rivero E, Rodríguez LG (2007). Incidence and risk factors for psoriasis in the general population. Arch Dermatol.

[21] Murdaca G, Colombo BM, Puppo F (2011). The role of Th17 lymphocytes in the autoimmune and chronic inflammatory diseases. Intern Emerg Med.

[22] Bazsó A, Szodoray P, Szappanos Á, Korda J, Pálfi P, Kiss E, Poór G (2015). Systemic autoimmune, rheumatic diseases and coinciding psoriasis: data from a large single-centre registry and review of the literature. Hindawi.

[23] Stojan G, Petri M (2018). Epidemiology of systemic lupus erythematosus: an update. Curr Opin Rheumatol.

[24] Korkus D, Gazitt T, Feldhamer I (2019). AB0788 Lupus co-morbidity in patients with psoriatic arthritis: a population-based case-controlled study. Ann Rheum Dis.

[25] Hu S, Lan C (2017). Psoriasis and cardiovascular comorbidities: focusing on severe vascular events, cardiovascular risk factors and implications for treatment. Int J Mol Sci.

[26] Khalid U, Ahlehoff O, Gislason GH, Kristensen SL, Skov L, Torp‐Pedersen C, Hansen PR (2014). Psoriasis and risk of heart failure: a nationwide cohort study. Eur J Heart Fail.

[27] Chen SK, Barbhaiya M, Fischer MA (2019). Heart failure risk in systemic lupus erythematosus compared to diabetes mellitus and general medicaid patients. Semin Arthritis Rheu.

[28] Varada S, Gottlieb AB, Merola JF (2015). Treatment of coexistent psoriasis and lupus erythematosus. J Am Acad Dermatol.

[29] Jamal S, Khan MZ, Kichloo A (2021). The effect of atrial fibrillation on inpatient outcomes of patients with acute pancreatitis: a two-year national inpatient sample database study. J Innov Cardiac Rhythm Manage.

[30] Edigin E, Kaul S, Eseaton PO (2020). Analysis of hidradenitis suppurativa hospitalizations: a report from the National Inpatient Sample database (Online ahead of print). J Am Acad Dermatol.

